# Systematic Identification and Expression Analysis of the Auxin Response Factor (ARF) Gene Family in *Ginkgo biloba* L.

**DOI:** 10.3390/ijms23126754

**Published:** 2022-06-17

**Authors:** Fangyun Guo, Wulai Xiong, Jing Guo, Guibin Wang

**Affiliations:** 1 College of Forestry, Nanjing Forestry University, Nanjing 210037, China; fangyunguo@njfu.edu.cn (F.G.); 15823686956@163.com (W.X.); jingguo@njfu.edu.cn (J.G.); 2 Co-Innovation Center for Sustainable Forestry in Southern China, Nanjing Forestry University, Nanjing 210037, China

**Keywords:** auxin response factors, developmental regulatory functions, *GbARF*s, expression profiles

## Abstract

Auxin participates in various physiological and molecular response-related developmental processes and is a pivotal hormone that regulates phenotypic formation in plants. Auxin response factors (ARFs) are vital transcription factors that mediate downstream auxin signaling by explicitly binding to auxin-responsive genes’ promoters. Here, to investigate the possible developmental regulatory functions of ARFs in *Ginkgo biloba*, through employing comprehensive bioinformatics, we recognized 15 putative *GbARF* members. Conserved domains and motifs, gene and protein structure, gene duplication, GO enrichment, transcriptome expression profiles, and qRT-PCR all showed that Group I and III members were highly conserved. Among them, *GbARF10b* and *GbARF10a* were revealed as transcriptional activators in the auxin response for the development of Ginkgo male flowers through sequences alignment, cis-elements analysis and GO annotation; the results were corroborated for the treatment of exogenous SA. Moreover, the *GbARF*s expansion occurred predominantly by segmental duplication, and most *GbARF*s have undergone purifying selection. The Ka/Ks ratio test identified the functional consistence of *GbARF2a* and *GbARF2c*, *GbARF10b*, and *GbARF10a* in tissue expression profiles and male flower development. In summary, our study established a new research basis for exploring Ginkgo *GbARF* members’ roles in floral organ development and hormone response.

## 1. Introduction

Auxin, the first and most abundant plant hormone discovered to be involved in growth and development, has been systematically studied in the developmental stages of different tissues and organs [1,2]. Indole-3-acetic acid (IAA) is a typical representative of auxin and is often found in rapidly developing organs, such as young stems and leaves, flowering organs, and seeds, as it mediates growth and, ultimately, determines the morphology of actively dividing and highly differentiated organs [3]. In this process, auxin response pathway regulates cell division, elongation, and differentiation, determining plants’ phenotypic characteristics and maintaining their growth [4]. Thus, auxin mediates organ differentiation, tissue development and growth rate at a macro level and contributes to their adaptation to dynamic environmental conditions [5].

The AUX/IAA protein, which encodes a primary auxin response gene, acts as a repressor of the auxin-responsive response that can be rapidly induced by auxin without generating an inducible protein that mediates the auxin signal initiation [4]. AUX/IAA acts on its own or forms a dimer with auxin response factors (ARFs) to inhibit auxin function, and this inhibition depends on the level of auxin distributed within the organ tissue [1]. ARFs constitute the foundation of auxin function, as ARFs contain a B3 domain that specifically binds to auxin-responsive elements; an ARF domain, which is related to transcriptional activation or to the repression of auxin-responsive genes; and a CTD domain that directly binds to AUX/IAA. These three domains are located in the N-terminal, the middle, and C-terminal of ARF proteins, respectively [6]. When the auxin level is high in plants, the transcriptional activity of auxin-responsive genes is activated by the ARFs, triggering an auxin response [7]. Conversely, ARFs bind directly to AUX/IAA to form dimers, which then bind to transcriptional repressors that prevent binding to the target gene, resulting in transcriptional inhibition of auxin response genes [1,8].

Arabidopsis is the most comprehensively and intensively studied model plant species with respect to the ARF family, which contains 23 members. Several of the ARF family members, such as *AtARF1* and *AtARF2* [9], *AtARF6* and *AtARF8* [10], and *AtARF7* and *AtARF19* [11], have been studied via mutant experiments, providing immense references for additional ARF family studies in other species. Subsequently, studies on ARFs in rice [12], tomatoes [13], and cotton [14] have advanced. However, with the exception of studies in poplar [15], apricots [16], apples [17], peaches [18], and eucalyptus [19], ARFs in woody plant species and other economically important tree species with relatively larger genomes have received less attention due to genomic resource barriers.

*Ginkgo biloba* is a typical gymnosperm of substantial economic value and is used for foliage, timber, and fruit production. There is only one species of Ginkgo in this Ginkgo genus, reflecting its unique botanical taxonomic status. Additionally, *G. biloba* is a relict plant and is dubbed as “the living fossil”, having existed for over 100 million years, which reflects its long and complex evolutionary history [20,21]. With the completion of the species genome sequence, it is no longer difficult to analyze and identify genes that play an important role in its survival, growth, development, and adaptation to dynamic environments [22]. In this study, 15 *GbARF* members were identified and analyzed based on the newly released Ginkgo genome, with references to other species’ sequences. The phylogenetic relationship, gene and protein structure, protein interaction networks, transcriptional and qRT-PCR profiling in tissues, and the gene ontology (GO) annotation were investigated to understand the regulatory roles of *GbARFs*. Through molecular cloning, relative expression analysis of the Ginkgo flower, and the test of responding to exogenous salicylic acid (SA), we identified and verified some candidate *GbARFs* that are involved in Gingko flower development and stimulation of SA, which provides a new foundation to further study the function of certain *GbARFs* in the Ginkgo flower development and in its responses to exogenous hormones.

## 2. Results

### 2.1. Identification of GbARF Proteins

A local BLAST search was performed to identify the ARF proteins in Ginkgo, and a total of 35 putative *GbARF* family members were identified. These 35 gene sequences were verified via the Conserved Domain Database (CDD) on the NCBI website, and 20 members without all three conserved domains (AUX_RESP, AUX/IAA andB3) were removed (Table 1). The genes were designated according to their phylogenetic relationship with Arabidopsis *AtARFs* (Table 1) [23]. The deduced full open reading frames (ORFs) ranged from 891 (*GbARF6a*) to 3834 bp (*GbARF2a*), with an average of 2665 bp. The putative protein sequences ranged from 296 (GbARF6a) to 1277 (GbARF2a) amino acid(aa). The predicted molecular weights (MW) varied from 33,086.80 (GbARF6a) to 141,221.98 Da (GbARF2a), and the isoelectric points (pI) varied from 5.46 (GbARF3) to 8.89 (GbARF6a). The grand average of the hydropathicity (GRAVY) values were all negative, indicating that these GbARF proteins were hydrophilic. Eleven of them were located in the nucleus, whereas GbARF2a, GbARF6a, GbARF2b, and GbARF4b were located in the plasma membrane, extracellular space, and the chloroplast, respectively.

### 2.2. Phylogenetic Analysis

**Figure 1:**
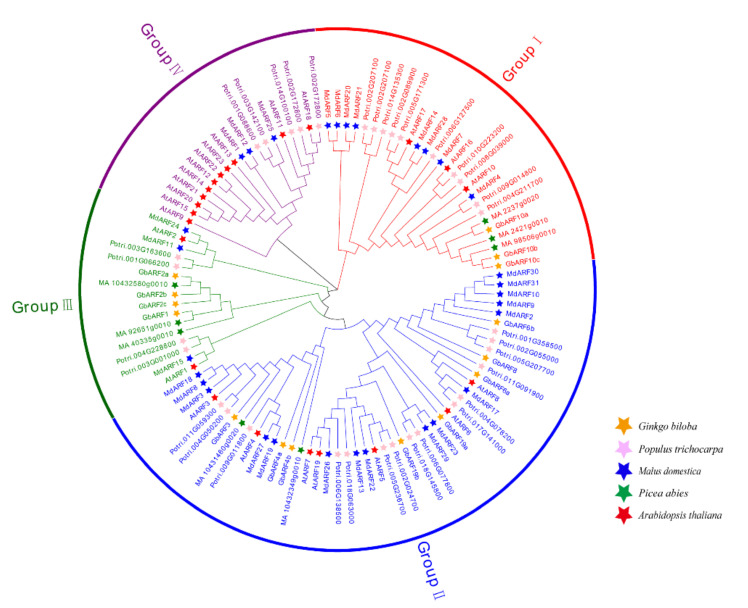
Phylogenetic tree of the ARF gene family in *Ginkgo biloba*, *Populus trichocarpa*, *Malus domestica*, *Picea abies*, and *Arabidopsis thaliana*. *GbARF* members of five species marked with different colored stars.

A phylogenetic tree was constructed comprising 23 identified ARF protein sequences from *Arabidopsis thaliana*, 37 from *Populus trichocarpa*, 31 from *Malus domestica*, and 8 from *Picea abies* (Figure 1). In reference to the evolutionary classification of ARFs in *Malus domestica* [17] and *Arabidopsis* *thaliana* [24], the ARF members in five species were divided into five Groups. The Ginkgo ARF members were distributed in Groups I–III. Group II contained eight members, which was the largest of the three Groups, while Group I and III contained three and four members, respectively. These seven members as well as GbARF3 and GbARF4b in Group II and the eight ARFs of *Picea abies* were on adjacent branches, indicating that the two gymnosperms are, phylogenetically, the most closely related. Moreover, the GbARFs being concentrated in Group III, indicated that duplication events may have occurred during the expansion of the *GbARFs* in the evolution of the Ginkgo genome.

### 2.3. Domain, Motif Identification, and Gene Structure Analysis

**Figure 2:**
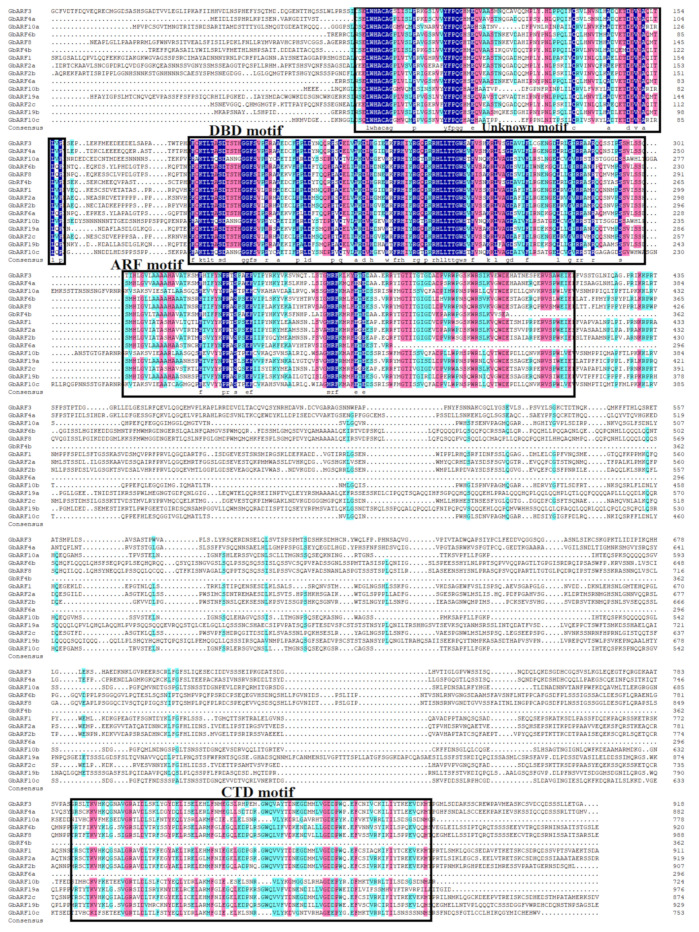
Multiple sequence alignment of GbARFs. Dark blue, light red, and light blue background indicates protein identity in 100%, 75%, and 50%, respectively. The DNA binding domain (DBD), auxin response factor (ARF) and C-terminal dimerization (CTD) domain regions were marked with black line boxes.

**Figure 3:**
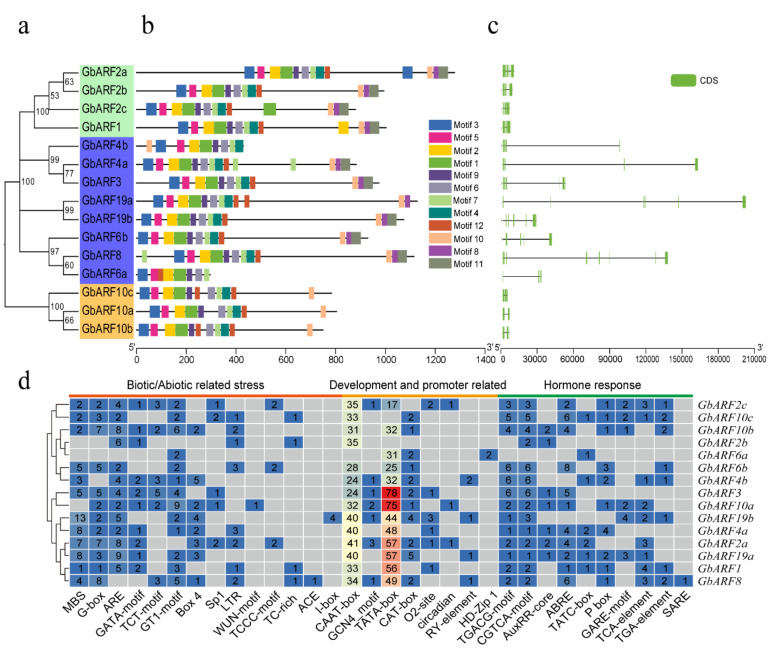
Phylogenetic tree, conserved motifs, gene structures, and regulatory elements of *GbARF* family. (**a**) Phylogenetic tree of GbARFs. (**b**) Conserved motifs in *GbARF*s. Different conserved motifs, numbers 1–12, are displayed in different colored boxes. (**c**) Exon-intron regions of *GBARFs*. (**d**) Numbers of cis-elements in *GbARFs*. Each gray box means that the gene does not contain following cis-element.

To further verify the conserved domains of the GbARF proteins, multiple sequence alignments were performed by DNAMAN 6.0 software (Figure 2). The results showed that, similar to the ARF proteins in other species [15,16,17], all GbARF*s* contained two conserved domains, DBD and ARF, which comprised more than 100 amino acids, respectively. Interestingly, Group I comprised GbARF10a, GbARF10b, and GbARF10c, and all of them contained QSL-rich regions, suggesting that they might act as activators to mediate AUX/IAA expression [25]. The other proteins had GSL-rich regions, suggesting that these GbARFs have suppressive properties [25]. A CTD domain was present in the remaining proteins, except for GbARF6a and GbARF4b, which may imply that these two genes function independently of auxin-responsive genes [18]. In the motif analysis, we found that all *GbARF* members had motif 1 and motif 2, at the N-terminus (Figure 3b), both of which are associated with DNA binding. The remaining members contained at least ten conserved motifs, while *GbARF6a* and *GbARF4b* had eight and nine motifs, respectively.

Overall, the distribution patterns (numbers and distribution position) of the motifs, exons, and introns for all the proteins were highly similar to their corresponding phylogenetic relationships (Figure 3a). For example, *GbARF1*, *GbARF2a*, *GbARF2b*, and *GbARF2c* all belong to Group III, these *GbARFs* had 4–22 exons and 13–21 introns; while *GbARF10b*, *GbARF10c*, and *GbARF10a* all belong to Group I, they had 3–4 exons and 2–3 introns (Figure 3c). The most visible was that the *GbARFs* with the blue background were members of Group Ⅱ, generally, the total lengths of their introns were much longer than those of the exons (Figure 3a,c), and the exon regions tended to be distributed at the beginning and end of the exon-intron sequences. These *GbARFs* exon–intron lengths were larger than those of the seven genes in Group I and III. These results indicated that the *GbARFs* were highly conserved within a single Group, which will help to further analyze their phylogenetic relationships and their regulatory function in developmental and environmental responses [26].

### 2.4. Analysis of cis-Elements in the Promoter of GbARFs

To better understand *GbARFs* functions and the probable regulatory pathways among them, an analysis of cis-acting elements in the promoters was conducted. The results showed that *GbARFs* contained a total of 33 cis-acting elements, all of them could be broadly clustered into the following three categories: biotic and abiotic stress response, plant development and promoter binding, and response to hormone induction (Figure 3d).

Among these elements, CAAT-box and TATA-box, two elements that bind to proteins, accounted for the highest proportion of all the elements in *GbARFs*. Except for *GbARF6a* and *GbARF8*, all the remaining genes in this family contained anaerobic-response elements (AREs), suggesting that most genes might play a role in the oxygen deprivation signaling response. Notably, 13 elements related to drought response, namely, MBSs, can bind with MYBs in response to the drought stress [27] and were present in the *GbARF19b* promoter, suggesting that *GbARF19b* might play a specific role in drought response. There were also elements identified that were related to meristem expression, and CAT-boxes were present in eleven of *GbARFs*’ promoters. Moreover, with the exception of *GbARF6a*, all the remaining genes contained TGACG or CGTCA motifs, showing a certain MeJA response for most genes. Abscisic acid response elements (ABREs) were present in 11 genes’ promoters, excluding *GbARF2b*, *GbARF6a*, *GbARF19b*, and *GbARF4b*, and this was the second most abundant element involved in hormone responses in the *GbARFs*.

### 2.5. Protein Tertiary Structure and Interaction Network Analysis

The GbARF proteins tertiary structures were predicted using sequences with more than 45% identity as templates, the results showed that all of the proteins mainly contained random coils (48.31–60.65%), alpha helixes (17.06–35.04%) and extended strands (13.89–23.50%) as part of their secondary structure (Table S3). The tertiary structures of GbARF10b, GbARF10c, and GbARF10a (belonging to Group I) were similar, as were those of GbARF1, GbARF2a, GbARF2b, and GbARF2c (belonging to Group III), while little consistency was found among the Group II members. These similarities and differences may imply the functional redundancy and divergence of *GbARFs* (Figure S1).

**Figure 4:**
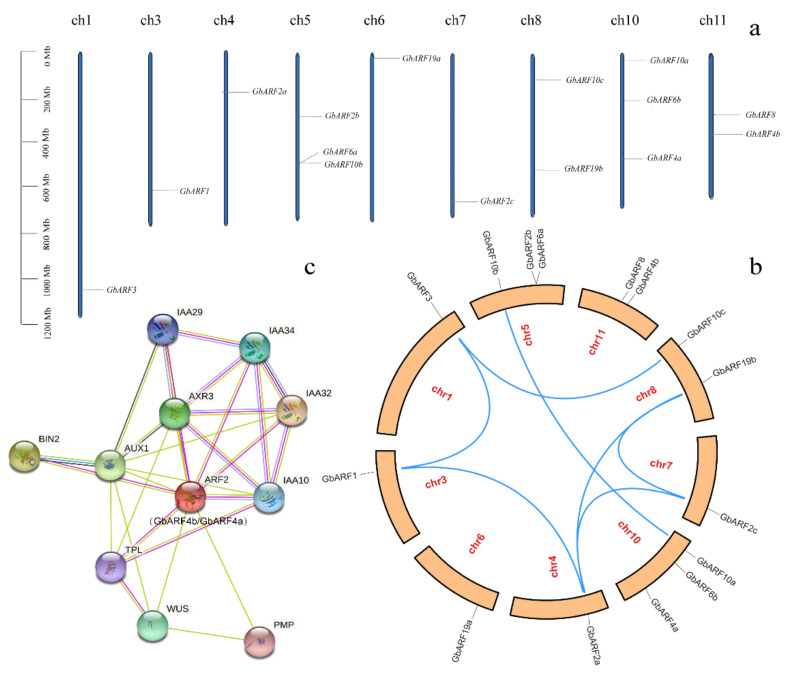
Physical chromosomal location, collinearity analysis and protein interaction of *GbARFs*. (**a**) Physical chromosomal location of *GbARFs*. (**b**) Duplicated relationship of *GbARF* genes. (**c**) A putative protein interaction network of *GbARF4b* and *GbARF4a*.

After the protein structure analysis, the orthologous ARF proteins in Arabidopsis were selected to further analyze the possible interaction networks to infer the biological function of the *GbARFs* in auxin signaling (Table S4). ARF4 (ortholog of GbARF4a), ARF6 (ortholog of GbARF19a), and ARF10 (ortholog of GbARF10a) all interact with MP and NPH4 (orthologs of GbARF19b) by encoding transcriptional receptors. These genes jointly participate in the modulation of auxin-dependent differential growth in Arabidopsis [28]. ARF10 is also involved in root cap development [29] and TIR1 has been shown to mediate the degradation of Aux/IAA transcriptional repressor proteins and regulate the development of leaves [29]. ARF8 (GbARF6a and GbARF8 orthologue) is a transcriptional activator of auxin gene expression that regulates stamen and gynoecium maturation by sensing auxin concentration [10] and interacts with BPEp and SHY2. BPEp is involved in the regulation of petal development, SHY2 functions as a repressor of early auxin response genes at a slow synthesis rate of IAA and is involved in root growth [10,29]. ARF2 (GbARF4b and GbARF4a orthologue) controls leaf senescence and floral organ abscission in Arabidopsis [9] (Figure 4c). Therefore, these *GbARFs* might function as their orthologs do in Aux/IAA transcription (Figure S2).

### 2.6. Chromosomal Location and Collinearity Analysis

The 15 *GbARFs* were unevenly distributed across 9 of the 12 Ginkgo chromosomes (Figure 4a). Three genes were located on chromosomes 5 and 10, separately, and only one was located on each of chromosomes 1, 3, 4, 6, and 7, respectively. A collinearity analysis showed a total of 7 fragmental duplication gene pairs were present on seven chromosomes (Figure 4b), among which, chromosomes 4 and 8 contained the most abundant duplicated pairs. The nonsynonymous substitution and the synonymous substitution (Ka/Ks) ratio of the *GbARF* pairs showed that only *GbARF3* and *GbARF1* were >1 (Table 2), while the remaining gene pairs were < 1, implying that segmental duplication is the main reason for the *GbARF* family’s expansion and that *GbARF3* and *GbARF1* have been subjected to positive selection pressure during the *GbARFs* evolution. These two genes may contribute to Ginkgo’s adaptation to various environmental conditions [30,31]. Additionally, the Ka/Ks values of four paralogous pairs (*GbARF1* and *GbARF2a*; *GbARF2a* and *GbARF19b*; *GbARF2c* and *GbARF19b*; *GbARF3* and *GbARF10c*) were >0.3, suggesting the possibility of functional divergence through duplication events [32]. Two paralogous pairs (*GbARF2a* and *GbARF2c*; *GbARF10b* and *GbARF10a*) had Ka/Ks values of <0.3, suggesting that their functions are conserved among paralogs [32]. Overall, those paralogous pairs whose Ka/Ks values were <1 reflect a stable evolutionary process because of the effect of the purifying selection.

### 2.7. GO Annotation of GbARF Family

**Figure 5:**
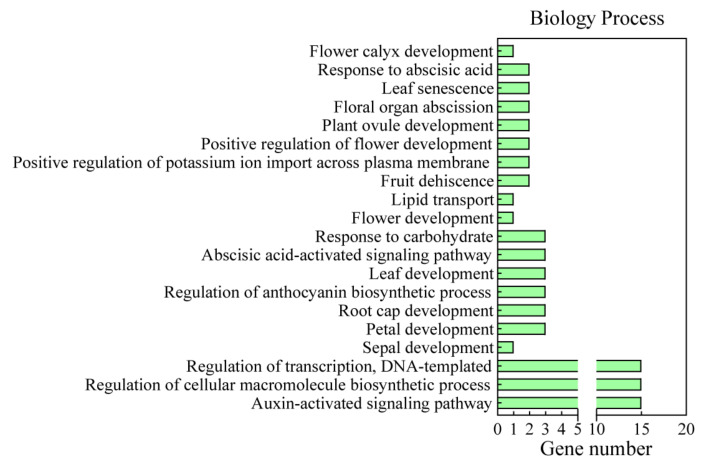
Annotation of biology process of GO analysis for *GbARFs*.

GO enrichment showed that all *GbARFs* were annotated to three broad term categories, including biological process, molecular function, and cellular component (Table S6). Each gene was annotated to the auxin-activated signaling pathway term in the biological process (Figure 5), demonstrating the accuracy of our *GbARF* family identification. Six genes (*GbARF10b*, *10c*, *10a*, *6b*, *2a*, and *2b*), were annotated to be involved in floral organ development (sepal, petal, and ovule), and *GbARF10a*, *10b*, *10c* were also annotated to the abscisic acid-activated signaling and the leaf development pathway, respectively (Figure 5). Molecular function mainly reflected the function of the *GbARF* products with DNA, protein and miRNA binding, and oxidoreductase activity. The cellular component explained that *GbARFs* were present in the nucleus.

### 2.8. GbARF Family Transcriptional Profiles

**Figure 6:**
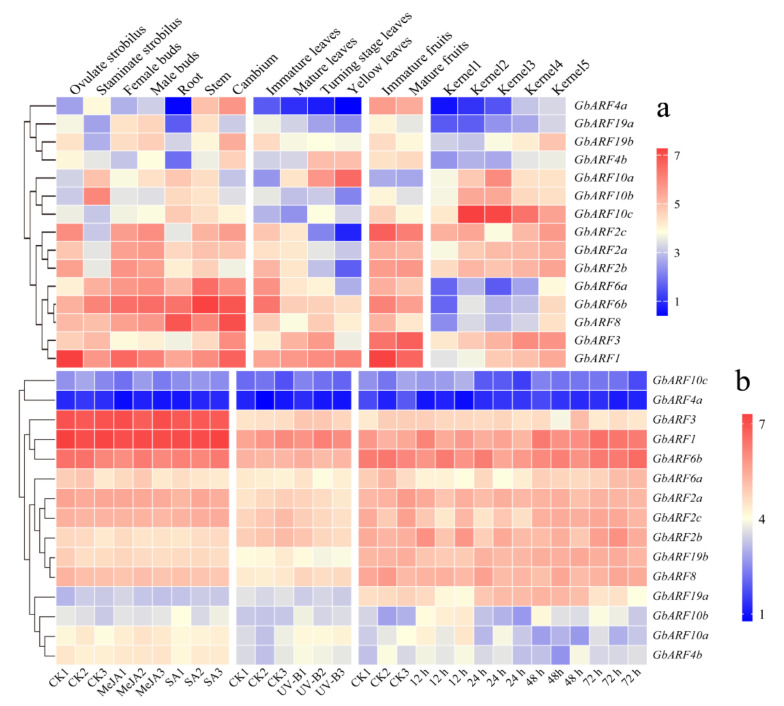
Expression profiles of *GbARFs* in different tissues, hormone induction and abiotic stress. (**a**) Tissue-specific expression of *GbARFs*. (**b**) Expression of *GbARFs* under hormone induction and abiotic stress. The FPKM, transcript abundance was converted to log_2_(FPKM+1) to construct heatmaps.

According to the expression pattern heatmap, we found most of the *GbARF* genes (*GbARF4a*, *GbARF19a*, *GbARF19b*, *GbARF2c*, *GbARF2a*, *GbARF2b*, *GbARF6a*, *GbARF3*) were specifically expressed in the stems, cambium, buds (both female and male) and immature fruits (Figure 6a). Moreover, the transcript levels of another class of members (*GbARF4a*, *GbARF19a*, *GbARF19b*, *GbARF10b*, *GbARF10c*, *GbARF2c*, *GbARF2a*, *GbARF2b*, *GbARF6a*, and *GbARF6b*) were gradually down-regulated with the tissue development. This was most notable in the leaves, as the levels reached their lowest at the yellow-leaf stage. Across the five stages of kernel development, with the exception of *GbARF2c*, the expressions of all the genes were distinctly up-regulated. These results suggested that certain *GbARFs* might play crucial roles in highly differentiated tissues, as well as in Ginkgo’s mature organs. Moreover, those genes that belonged to the same group, such as *GbARF10b*, *GbARF10c*, and *GbARF10a* (Group I); and *GbARF2a*, *GbARF2b*, and *GbARF2c* (Group III), exhibited similar expression patterns, which corroborated the conservative function of *GbARF10b*, *GbARF10a* and *GbARF2a*, *GbARF2c* in syntenic analysis. In contrast, most Group II members showed a varied main expression, such as *GbARF4a*, *GbARF19a*, *GbARF6b*, *GbARF8*, and *GbARF3*.

Under MeJA and SA induction, the *GbARFs* were not responsive (Figure 6b). There were no visible expression differences between the treatments and the controls (CKs). However, the expressions of *GbARF3*, *GbARF8*, *GbARF10a*, and *GbARF4b* were increased after UV-B exposure, reflecting a certain functional activity in response to light signals. After PEG-6000 treatment, *GbARF6a* and *GbARF19a* were downregulated, compared with their CKs at 12 h, respectively, after which they gradually recovered over a period of 48 h. In contrast, *GbARF10b*, *GbARF10a*, and *GbARF4b* were upregulated at 12 h, while their levels were consistent with those of the CK, up to 24 h. *GbARF10b* was upregulated again at 48 h, while *GbARF10a* and *GbARF4b* were continuously downregulated. Taken together, the results suggest that these five *GbARFs* may be involved in temporal, dynamic biological response process to drought.

### 2.9. Quantitative Real-Time PCR(qRT-PCR) Verification of Tissue-Specific Expression

**Figure 7:**
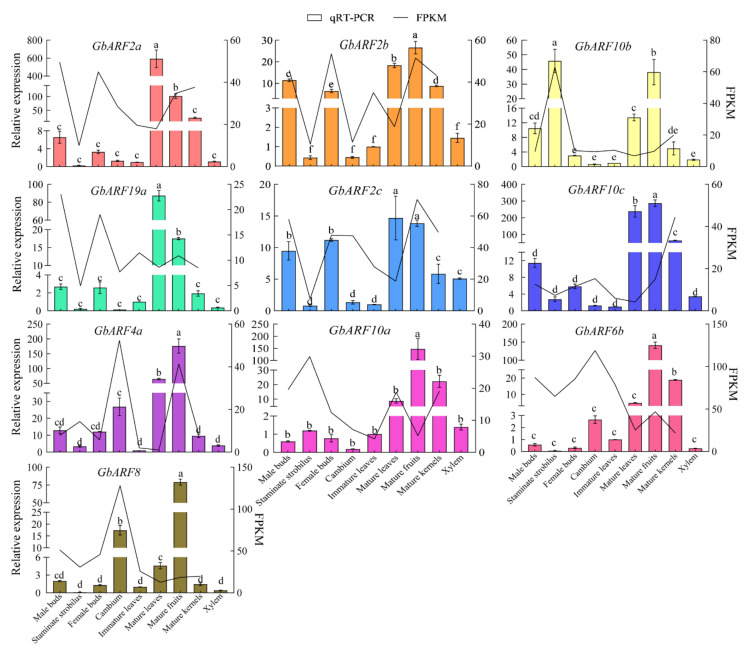
qRT-PCR validation for 10 selected genes. The colored vertical bars of each gene represent relative expression level in qRT-PCR and the black folds show FPKM value in transcript level. Relative expression was indicated as means ± standard deviation (SD). Different letters denote significant differences at 0.05 level by Duncan’s multiple range test.

To verify the expression patterns and potential regulatory roles of the *GbARF* family in different tissues, 10 genes that had been previously predicted for biological functions were selected for qRT-PCR assays. In general, the expression levels of 10 genes were consistent with the fragments per kilobase per million (FPKM) values, except for the levels of the mature leaves and fruits(Figure 7), this inconsistency was probably due to the different materials used in this test and the transcriptome sequencing test. The expression of *GbARF2b* and *GbARF2c* in male and female buds, mature leaves, and fruits were significantly higher than in other tissues, supporting the GO prediction that *GbARF2b* is involved in fruit dehiscence and the positive regulation of flower development. *GbARF2a*, *GbARF2b* and *GbARF2c* showed priority expression in mature leaves in a relative level; considering that *PtARF2.1*, as a *GbARF2* homolog, has been reported to be involved in leaf developmental regulation and lignin biosynthesis in poplar, it was mainly expressed in leaves [26]. Therefore, here, the expression patterns of three *GbARF2s* might indicate that these *GbARF2s* were involved in the consistent developmental regulation as *PtrARF2.1*. The high expression of *GbARF10b* in staminate strobilus was in agreement with its predicted function in the biological process, it was allocated to the term of petal and calyx development. In addition, *GbARF4a*, *GbARF6b*, and *GbARF8* were not only highly expressed in mature leaves, fruits, and kernels, but were also significantly up-regulated in cambium, compared to other tissues. This phenomenon was different from the other genes and supported the transcriptional profile above.

### 2.10. GbARF6a, GbARF10b, and GbARF10a Cloning and Expression Profiles of GbARFs in Flower Development

**Figure 8:**
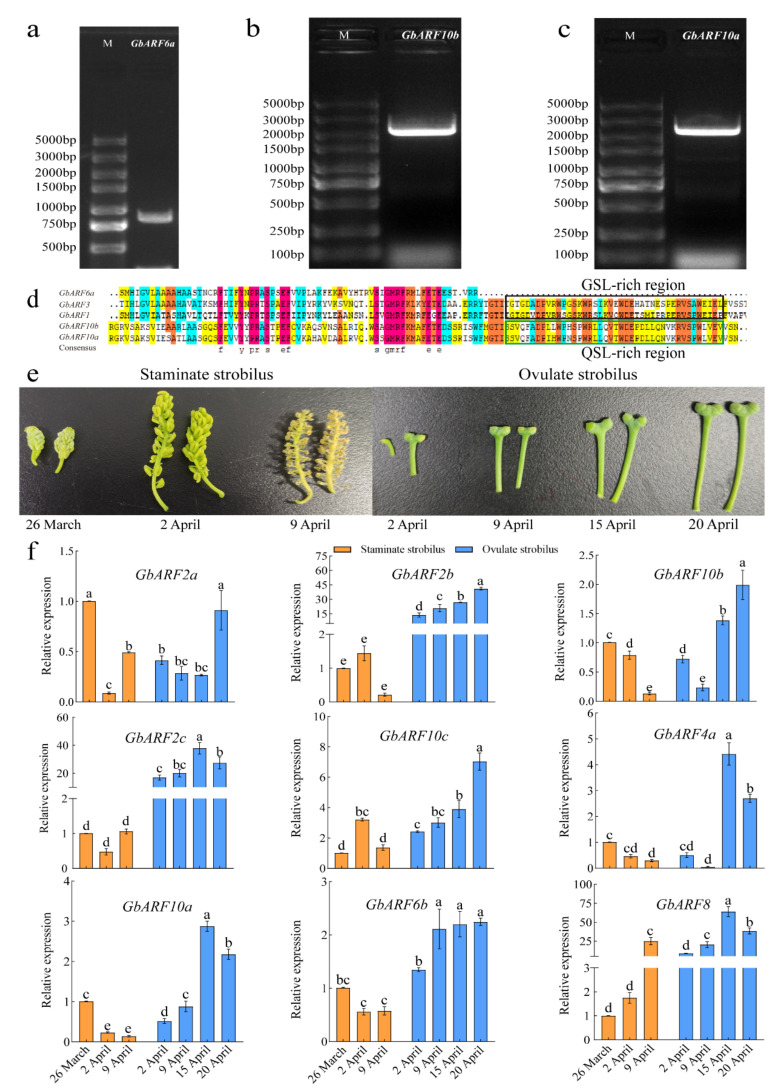
PCR amplification of *GbARF6a* (**a**), *GbARF10b* (**b**), *GbARF10*a (**c**) and identification of ARF domain (**d**). (**e**) Developmental stages of staminate strobilus and ovulate strobilus. (**f**) Relative expression of 9 *GbARFs* during male and female flower development in Ginkgo. Orange and blue vertical bars represent staminate strobilus and ovulate strobilus, respectively, expression levels were indicated as means ± standard deviation (SD), different letters denote a significant difference of 0.05 in Duncan’s multiple range test.

*GbARF6a*, *GbARF10b* and *GbARF10a* were used as candidates for molecular cloning to validate the putative protein-coding regions. According to the GO functional annotation and the tissue-specific expression, cDNA from early developmental staminate strobilus (collected on 26 March 2022) were used as templates to amplify the full-length ORFs of three genes. The amplified products are shown in Figure 8a–c, sequencing results suggested that the protein coding regions of the three genes were fully consistent with the previous genomic identification, and the nucleic acid coding region of *GbARF6a* was only 860 bp, without CTD domain, reflecting that it is not dependent on the Aux/IAA for its function. The ARF domain ends of *GbARF10b* and *GbARF10a* contained more Q than the other members (Figure 8d), revealing their transcriptional activation in auxin signaling [25].

We further selected nine *GbARFs* to analyze their possible roles in Ginkgo flower development by comparing the expression profiles during three development stages of staminate strobilus and four development stages of ovulate strobilus (Figure 8e). *GbARF2b* was involved in ovule development and its comparative expression in ovulate strobilus was at least nine-fold higher than in male flowers (staminate strobilus), which reached the highest on 20 April (Figure 8f). Meanwhile, *GbARF10b*, *GbARF10c* and *GbARF10a* were all assigned to the term petal and sepal development. They had higher expression at two later stages (15 and 20 April) of ovule development than staminate strobilus. This indicates that these three genes play an integral role in female flowers’ maturation. Notably, the expression variation of *GbARF2a* in staminate strobilus was consistent with that of *GbARF2c*, as also *GbARF10b*, *GbARF4a*, *GbARF10a*, and *GbARF6b*.The latter four genes had the highest expression at the early stage of male flower development (26 March), and their expression gradually decreased on 2 April and 9 April. Overall, nine selected *GbARFs* were preferentially expressed in female flowers rather than the male flowers, especially in the latter two stages of development.

### 2.11. Expression of GbARFs in Response to Exogenous Salicylic Acid (SA)

**Figure 9:**
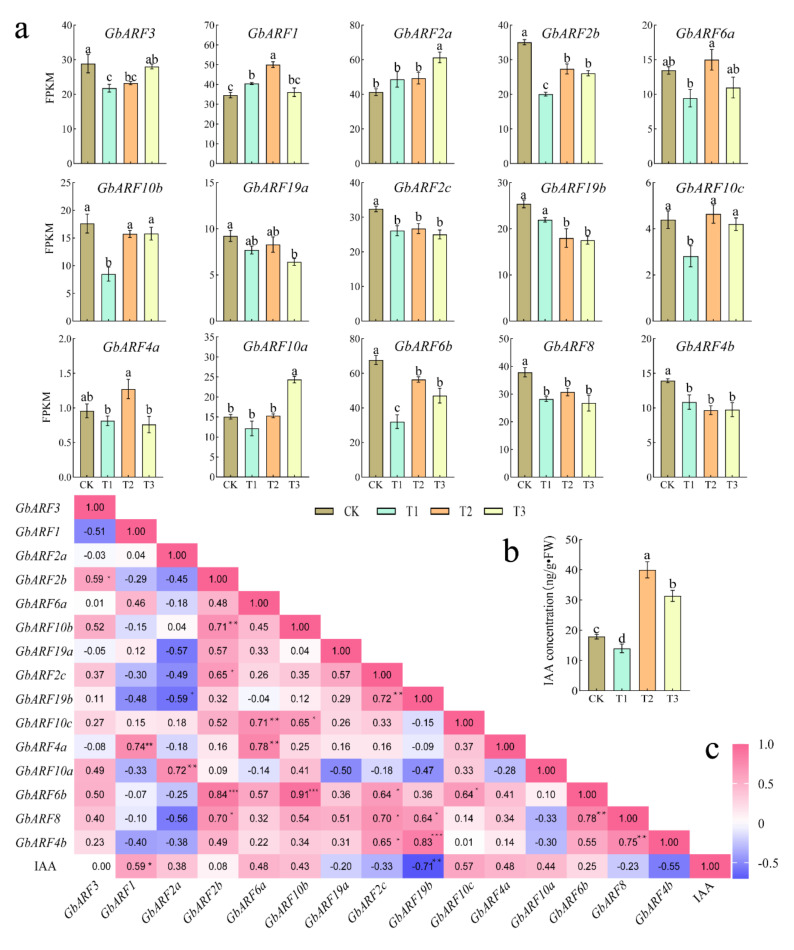
Expression profiles of *GbARFs* and the correlation with IAA concentration under SA treatment. (**a**) FPKM values of *GbARFs* (**b**) IAA Concentration. (**c**) Correlation heat map in *GbARFs* expression level and IAA response to SA. FPKM values were showed as mean means ± standard error (SE). CK, T1, T2 and T3 in (**a**) represent exogenous SA concentration of 0, 1, 2 and 3 mmol/L, respectively. Letters in Figure (**a**,**b**) represent significant differences at the 0.05 level, and * in Figure (**c**) means the 0.05 significant level, ** means the 0.01 level, and *** means the 0.001 level.

In the exogenous SA treatment assay, with the exception of *GbARF6a* and *GbARF4a*, the remaining genes all showed significant response (Figure 9a). Among them, members containing more SA cis-elements showed the following two expression trends overall: (1) *GbARF1*, *GbARF2a*, and *GbARF10a* were significantly up-regulated than the CK, and (2) *GbARF2c*, *GbARF19b* and *GbARF8* were down-regulated. In addition, *GbARF6b* had a significant correlation in expression level with *GbARF2b* and *GbARF10b*, respectively, as was also the case between *GbARF4b*, *GbARF19b* and *GbARF8*, and between *GbARF2b* and *GbARF10b*, respectively. More interestingly, the expression of *GbARF1*, *GbARF10b*, *GbARF10c* and *GbARF10a* were positively correlated with the IAA concentration under SA stimulation (Figure 9c), which verified our earlier identification of them as activators. *GbARF10b*, *GbARF10c* and *GbARF10a* mediated the expression of IAA-related genes, and might be involved in the transportation of endogenous IAA induced by exogenous SA, resulting in new growth and development [33].

## 3. Discussion

Auxin is an essential hormone for plant development, from influencing embryonic differentiation to causing organ abscission and even the survival of the entire plant. These biology processes cannot be initiated without auxin induction. ARFs constitute a class of transcription factors that are associated with growth and stress responses and mediate the development of various tissues in plants [9,10,11,12]. Previous studies have suggested that the number of ARFs in different plants is not directly related to their genome size, which varies widely among species. For instance, there are 50 ARF genes in *Osmanthus fragrans* [34], 17 in physic nut [35], 31 in apple [17], 26 in *Brachypodium distachyon* [36], 17 in melon [37], 17 in peach [18], 19 in *Vitis vinifera* [38], 51 in soybean [25], and 28 in chickpea [32]. *Ginkgo biloba*, is the only broad-leaved gymnosperm that exists, the members and biological features of *GbARF* family still remain unknown.

In this study, based on the recent release of more than nine Gb Ginkgo genomes, a total of 15 *GbARF* members were identified. The CTD domain for two members (*GbARF6a* and *GbARF4b*) was truncated, which accounted for 13.3% of all the genes, this percentage was lower than that in *Osmanthus fragrans* (18%) [34], maize (30.6%) [39], melon (23.5%) [37] and Medicago (54.2%) [40], suggesting that CTD is a key region for the function of *GbARF*s. The 15 members were grouped into three groups according to their homology with the ARFs in Arabidopsis, Populus, *Malus domestica* and *Picea abies*: Groups I-III. Of which, *GbARF6a*, *GbARF6b* and *GbARF8*(that belong to Group II) were identified as homologs of ARF6/8-like in Ginkgo, which was previously reported in other study [23]. The protein sequence identity of these three members and of ARF6/8-like were 100%, 70.2% and 84.9%, respectively. *GbARF6b* and *GbARF8*, as two new ARF6/8-like homologs, were recognized in Ginkgo, which will facilitate the study of their regulatory functions. Overall, the identifications of motifs, intron–exons, cis-acting elements, and protein tertiary structures indicated that the organization of the *GbARFs* were fairly conserved within a given group, supporting their classification and evolutionary relationships among the members and implying that they have a redundant function in each subgroup. Subsequently, *GbARF10b*, *GbARF10a* and *GbARF10c*, as the members of Group I, were verified as transcriptional activators due to the Q enrichment at the end of the ARF domain by gene cloning and sequence alignment [25]. Furthermore, *GbARF10a* and *GbARF8* were as the orthologs of *AtARF10* and *AtARF8*, respectively. *GbARF10a* exhibited predominant expression in yellow leaves and *GbARF8* was highly expressed in flowers and fruits in transcriptional and relative expression profiles. Studies have shown that together with TIR1, *AtARF10* coregulates leaf development and root cap formation [41], and that *AtARF8* interacts with BIGPETALp (BPEp) to regulate flower fertilization and fruit development in Arabidopsis [10]. Therefore, these predominant expressions may suggest the putative functions of *GbARF10a* and *GbARF8* in organs’ development.

It has been considered that the cis-acting elements positively correlate with the regulatory functions of genes [42,43]. In our work, eight *GbARFs* had AuxRR core or TGA elements that specifically bind to downstream target Aux/IAA [29]. *GbARF10b* was found to have four of these elements in its promoter, as the most among the eight genes. *GbARF10b* and *GbARF10a* contained more elements associated with light response than those of the remaining *GbARFs*. The transcript abundance and relative expression were analyzed to further recognize these genes’ roles. *GbARF10a* was confirmed to have more sensitive transcript abundance after UV-B and PEG-6000 treatment, and members of Group I and III showed the same pattern in their tissue-specific expressions, demonstrating previous prediction that they share consistent a function in tissue development. In Ginkgo flowers, *GbARF10b*, *GbARF4a*, *GbARF10a*, and *GbARF6b* showed the highest expression in the early stages of staminate strobilus development (26 March), and then gradually decreased to the lowest level after 2 weeks. This suggests that these minimum expressions of the four genes were related to the late development of the staminate strobilus, the typical developmental phenotype for which is that after the pollen sacs are opened to release mature pollens, the staminate strobilus are close to wilting. This result is in line with down-regulated *LcARFs* in the treatment of litchi fruit abscission [44]. On the other hand, the high expression level of nine genes at the latter two stages of ovule verified their biological functional predictions, which stated that they were involved in ovule development and fertilization. Thus, we can speculate that most of the *GbARFs* play important mediating roles in the IAA-induced initial development of male flowers and, later, of female flowers. However, further experimentation is needed to verify our hypothesis.

Under exogenous SA treatment, *GbARF1*, *GbARF10c* and *GbARF10a* were positively correlated with the IAA concentration, respectively. These results were in agreement with those reported for ARF in *Osmanthus fragrans* [34] and tomatoes [45], suggesting that three *GbARFs* are positively involved in IAA signaling under exogenous SA stimulation. At the same time, the expression of *GbARF10c* was also positively correlated with *GbARF6a* and *GbARF10b*.This finding is also in agreement with that of soybean [25], chickpea [32], and maize [39], respectively, where some ARFs functional redundancy may be due to segmental duplication. Overall, most of the *GbARFs* showed a down-regulation expression under T1 treatment, followed by up-regulation in higher concentrations of exogenous SA. This can be explained that slight SA stimulation weakened the IAA action because of co-regulation among endogenous phytohormones [45], while the stronger induction of exogenous SA in T2 and T3 groups activated the IAA metabolic pathway [45].

## 4. Materials and Methods

### 4.1. Identification and Sequence Analysis of GbARFs

The recent Ginkgo genome-wide data were obtained from Liu H.L. [20]. The published full-length ARF protein sequences of *Arabidopsis thaliana* [24], *Populus trichocarpa* [15], *Picea abies* and *Malus domestica* [17] were obtained from the Plant Transcription Factor Database (PTFD) (http://planttfdb.gao-lab.org/, accessed on 10 December 2021). First, the ARF protein sequences of the above four species were used as query sequences to search for ARF members in *Ginkgo biloba*, via local BLASTP (https://ftp.ncbi.nlm.nih.gov/blast/executables/blast+/LATEST/, accessed on 20 December 2021), with an E-value ≤ 10 − 5. Second, the sequences of all putative members were merged from four searches, and the repetitive sequences were removed. The online Conserved Domains Database (CDD: https://www.ncbi.nlm.nih.gov/cdd, accessed on 23 December 2021) was used to detect AUX_RESP, AUX/IAA, and B3 conserved domains, members lacking more than two of these domains were deleted [24]. The physicochemical properties of GbARFs were subsequently predicted using ExPASy (http://web.expasy.org/protparam/, accessed on 28 December 2021), and subcellular localization of each *GbARF* was determined using WoLF PSORT (http://psort.hgc.jp/, accessed on 1 January 2022).

### 4.2. Phylogenesis, Protein Conserved Domain, Motif and Gene Structure Analysis

ARF protein sequences from *Arabidopsis thaliana*, *Populus trichocarpa*, *Picea abies*, *Malus domestica* and *Ginkgo biloba* were used to construct a phylogenetic tree via MEGA 7.0 software (https://www.megasoftware.net/, accessed on 6 January 2022) based on the maximum likelihood method, with 1000 bootstrap replications [46]. Multiple sequence alignment was performed using the ClustalW program of MEGA 7.0. The tree was then visualized using the online tool EvolView (http://www.evolgenius, accessed on 10 January 2022).

To confirm the highly conserved domains among *GbARF* members, sequence alignments were conducted by DNAMAN software [46]. Twelve conserved motifs were searched with the default parameters to distinguish the organization of *GbARF*s using the online website, Multiple Em for Motif Elicitation (http://meme.nbcr.net/meme/intro.html, accessed on 13 January 2022). The genome annotation gff3 file was used to extract exon-intron sequences from the *GbARF* genes, after which TBtools [47] analyzed their structure and constructed diagrams.

### 4.3. Protein Tertiary Structure, Cis-Acting Element and Interaction Network Analysis

The tertiary structures of the GbARFs were predicted using SWISS-MODEL (https://swissmodel.expasy.org/, accessed on 13 January 2022), based on template sequences that had more than 45% identity with GbARFs. We submitted the 2000 bp sequence upstream of the start codon of *GbARF* genes in the Ginkgo genome to PlantCARE (http://bioinformatics.psb.ugent be/webtools/plantcare/html/, accessed on 14 January 2022) to predict their cis-regulatory elements and then displayed the element locations by TBtools for subsequent analysis [47]. The GbARF protein interaction network was analyzed using String (https://string-db.org/, accessed on 15 January 2022) based on the highest-scoring proteins identified via BLAST of TBtools.

### 4.4. Chromosomal Location and Synteny Analysis

All *GbARF* genes were mapped to the Ginkgo chromosomes according to the data in the gff3 file, and gene duplication analysis of the paralogous genes was performed using TBtools [47]. Briefly, the “Gene Location Visualize” component of the GFF tool was used to construct a diagram of the physical location of the genes on the chromosomes. The protein sequences were matched using the subroutine “Two Sequence Files” of BLAST to perform pairwise alignment of the GbARFs. The E-value was set as 1 × 10^−5^. Both NumOfHits and NumOfAligns were set to 5, and the output file was obtained for further analysis. Finally, the gff3 and alignment files were utilized to analyze the collinear relationship using the Quick MCScanX Wrapper program [47,48]. The advanced Circos tool was subsequently used to display pairwise relationships. The DnaSP 6.0 (http://www.ub.edu/dnasp/, accessed on 17 January 2022) software was used to calculate nonsynonymous substitution/synonymous substitution (Ka/Ks) ratios for *GbARF* paralogous pairs.

### 4.5. GO Annotation Analysis

The online tool Protein ANNotation with the Z-scoRE server (PANNZER) (http://ekhidna2.biocenter.helsinki.fi/sanspanz, accessed on 21 March 2022) was utilized to describe comprehensive annotation of gene function and gene products for all *GbARF* members.

### 4.6. Transcription Analysis of GbARF Family Genes

The publicly available RNA raw sequencing (RNA-seq) data in the European Nucleotide Archive (ENA) database (https://www.ebi.ac.uk/ena/browser/view/,accessed on 20 October 2021) were downloaded (Table 3), including from strobilus, buds, roots, stems, cambium, leaves, and fruits in different development stages as well as the kernels. Additionally, the transcriptional profiles also contained data in response to induction of methyl jasmonate (MeJA), salicylic acid (SA), UV-B and simulated drought stress caused by polyethylene glycol (PEG)-6000. We also conducted a trial of exogenous SA spraying on the leaves of Ginkgo seedlings, 12 samples from which were sequenced by Shanghai Majorbio Bio Co., Ltd. (Shanghai, China) using Illumina NovaSeq 6000. After evaluating the raw data by FastQC (https://www.bioinformatics.babraham.ac.uk/projects/fastqc, accessed on 30 October 2021), the low-quality reads and adapter sequences were removed by Trimmomatic [49]. The clean reads were mapped to the newest Ginkgo genome by STAR [50], and the fragments per kilobase per million mapped reads (FPKM) values were then calculated by RSEM [51]. Finally, the transcript abundance of GbARFs were converted to log_2_(FPKM + 1) values, and an expression heatmap was constructed with GraphPad Prism 8.0.

### 4.7. GbARF6a, GbARF10b, and GbARF10a Cloning

Total RNA from young staminate strobilus (collected on 26 March) were used to obtain cDNA using a reverse transcription kit (Monad Biotech Co., Ltd., Nanjing, China). The full length CDS was amplified with three pairs of specific primers (Table S7). The PCR procedure was as follows: 95 °C for 3 min, followed by 35 cycles of 95 °C for 15 s, 60 °C for 15 s, 72 °C for 52 s in *GbARF6a* and for 130 s in *GbARF10b* and *GbARF10a*, finally at 72 °C for 5 min. The amplified products were injected in 1% agarose gel to move the positive side of the gel bath with voltage of 120 V and the quality of products were observed on a gel imager. The gel blocks containing the target genes were purified using FastPure^®^ Gel DNA Extraction Mini Kit (Vazyme Biotech Co., Ltd., Nanjing, China), then ligated into the clone vector pClone007 Blunt Simple Vector (Tsingke, Nanjing, China), and transferred into DH5α strain (Tsingke, Nanjing, China). The resuscitation solution was spread on LB solid medium, then put in 37 °C incubator to dark culture for 16 h, the positive clones were identified and sequenced to verify their genomic sequences.

### 4.8. qRT-PCR Expression of GbARFs

Staminate strobili, ovulate strobili, and buds at different developmental stages were harvested from ten Ginkgo trees (five females and five males) that were approximately 20 years old, located in the Nanjing Forestry University campus. Specifically, the female and male buds, as well as the young staminate strobilus were separately harvested on 26 March 2022. After this the middle and late developmental staminate strobili were taken after 1 week (2 April) and 2 weeks (9 April), respectively. The ovulate strobili were harvested on 2 April, 9 April, 15 April, and 20 April 2022, respectively. Other tissues were all collected from female Ginkgo trees. The immature leaves were harvested in June 2021, and the mature leaves, cambium and xylem, fruits, and kernels were harvested in October 2021. Three biological replicates of each tissue from five females/males were rapidly frozen in liquid nitrogen for total RNA extraction. The extraction procedure was performed according to the instructions of the E.Z.N.A.^®^ Plant RNA Kit (Omega, Nanjing, China). After the total RNA was tested for concentration and purity, the cDNA was synthesized using MonScript™ RTIII All-in-One Mix with dsDNase (Monad, Nanjing, China), and 1ul of cDNA was used in MonAmp™ SYBR^®^ Green qPCR Mix (Monad, Nanjing, China) to react PCR with an Applied Biosystems^®^ 7500 Real Time PCR System (Thermo Fisher Scientific Inc., Waltham, MA, USA). PCR program was set as 30 s at 95 °C, 40 cycles of 95 °C for 10 s, and 60 °C for 15 s, and the parameters of melting curves were default by instrument that were collected before. The *GAPDH* gene was used as an internal control to analyze the relative expression level for *GbARF* members by the 2^–ΔΔCt^ method [62].

### 4.9. Plant Materials and Exogenous SA Treatments

Two-year-old Ginkgo seedlings provided the materials for this experiment, and three treatments of exogenous SA gradient concentration were set as follows: 1 mmol/L (T1), 2 mmol/L (T2) and 3 mmol/L (T3), with 0 mmol/L concentration as the control (CK). A total of 25 seedlings were sprayed for each treatment. The spraying trials were conducted four times:1 June, 8 June, 22 June, and 29 June 2019, Twenty days after completion of the 4 spray times, the mature leaves were collected for transcriptome sequencing and IAA concentration determination. The raw sequencing data have been deposited in the China National Center for Bioinformation (CNCB) database (accession no. PRJCA009411). One biological replicate of each treatment was obtained from eight seedlings, for a total of three replicates. IAA concentration was determined using an enzyme-linked immunoassay kit from the Institute of Hormone Research, China Agricultural University, and the test procedure was referenced to Sheng et al. [33].

### 4.10. Statistical Analysis

All samples used in this experiment were three biological replicates, the correlation and one-way analysis of variance (ANOVA) (Duncan’s test) were analyzed using GraphPad Prism 8.0 software (https://www.graphpad.com/, accessed on 1 August 2021).

## 5. Conclusions

In summary, we recognized 15 *GbARF* members by genome-wide identification in *Ginkgo biloba* and grouped them into Groups I–III based on phylogenetic analysis. Multiple analyses including sequence alignment, structure characteristics, and GO annotation, all suggested that the *GbARFs* that were part of the same Group were highly conserved in structure and biological function. Subsequently, transcriptome, gene cloning, and qRT-PCR were conducted to investigate their possible regulatory roles in responding to flower development and to the stimulation of exogenous SA. *GbARF10b* and *GbARF10a* were verified as candidate genes that acted as transcriptional activators of the IAA signaling involved in Ginkgo male floral organ development and in the response of exogenous SA. This study provides insight into the regulatory functions of certain *GbARFs* associated with Ginkgo floral organ development and exogenous hormones, which will contribute to further research on the mechanisms of regulatory functions for *GbARFs* in Ginkgo.

## Figures and Tables

**Table 1:** The protein characteristics of GbARFs in Ginkgo.

Gene Name	Gene ID	ORF	AA	MW (Da)	pI	GRAVY	Subcellular Localization
*GbARF3*	evm.model.chr1.3000	2922	973	108,130.40	5.46	−0.458	Nuclear
*GbARF1*	evm.model.chr3.2036	3009	1002	110,896.65	6.63	−0.530	Nuclear
*GbARF2a*	evm.model.chr4.677	3834	1277	141,221.98	6.5	−0.335	Plasma membrane
*GbARF2b*	evm.model.chr5.1456	2982	993	109,968.78	6.62	−0.516	Chloroplast
*GbARF6a*	evm.model.chr5.1462	860	285	33,086.80	8.89	−0.242	Extracellular
*GbARF10b*	evm.model.chr5.854	2250	749	82,844.92	6.64	−0.476	Nuclear
*GbARF19a*	evm.model.chr6.93	3384	1127	126,560.12	6.06	−0.516	Nuclear
*GbARF2c*	evm.model.chr7.1996	2640	879	97,850.61	5.59	−0.615	Nuclear
*GbARF19b*	evm.model.chr8.1790	3222	1073	121,038.59	6.36	−0.692	Nuclear
*GbARF10c*	evm.model.chr8.618	2352	783	86,475.31	7.60	−0.447	Nuclear
*GbARF4a*	evm.model.chr10.1507	2649	882	97,626.73	5.83	−0.469	Nuclear
*GbARF10a*	evm.model.chr10.178	2412	803	88,729.16	6.44	−0.512	Nuclear
*GbARF6b*	evm.model.chr10.810	2790	929	103,431.84	6.13	−0.506	Nuclear
*GbARF8*	evm.model.chr11.851	3345	1114	123,501.21	7.34	−0.482	Nuclear
*GbARF4b*	evm.model.chr11.970	1290	429	48,215.21	8.70	−0.209	Chloroplast

**Table 2:** Ka (nonsynonymous), Ks(synonymous), and Ka/Ks ratio of duplicated GbARF genes.

Gene 1	Gene 2	Ka	Ks	Ka/Ks(ω)	Selection	Duplication Mode
*GbARF3*	*GbARF1*	3.18237	2.30424	1.38109	Positive	Segmental
*GbARF1*	*GbARF2a*	0.28025	0.89312	0.31379	Purifying	Segmental
*GbARF2a*	*GbARF2c*	0.18970	0.86208	0.22005	Purifying	Segmental
*GbARF2a*	*GbARF19b*	0.93854	1.34106	0.69985	Purifying	Segmental
*GbARF10b*	*GbARF10a*	0.14941	0.96258	0.15522	Purifying	Segmental
*GbARF2c*	*GbARF19b*	0.72979	0.89579	0.81469	Purifying	Segmental
*GbARF3*	*GbARF10c*	0.84295	1.16164	0.72566	Purifying	Segmental

**Table 3:** List of RNA-Seq data in GBARF family of Ginkgo.

Project Number	Sample Type	Library	Platform	Reference
PRJNA289172	Ovulate strobilus	Paired end	Illumina HiSeq 2500	[52]
PRJNA289172	Staminate strobilus	Paired end	Illumina HiSeq 2500	[52]
PRJNA289172	Female and male buds	Paired end	Illumina HiSeq 2500	[52]
PRJNA373812	Roots	Paired end	Illumina HiSeq 4000	[53]
PRJNA473396	Stems	Paired end	Illumina HiSeq 4000	[54]
PRJNA488475	Cambium	Paired end	Illumina HiSeq 2000	[55]
PRJNA473396	Immature leave	Paired end	Illumina HiSeq 4000	[54]
PRJNA517218	Mature leave	Paired end	HiSeq X Ten	[56]
PRJNA578374	Turning stage and yellow leave	Paired end	Illumina HiSeq 4000	[57]
PRJNA473396	Immature and mature fruits	Paired end	Illumina HiSeq 4000	[54]
PRJNA292849	Kernel1–5	Paired end	Illumina HiSeq 2000	[58]
PRJNA553587	Treatment of MEJ	Paired end	HiSeq X Ten	[59]
PRJNA598887	Treatment of SA	Paired end	HiSeq X Ten	[59]
PRJNA595103	Treatment of UV-B	Paired end	Illumina HiSeq 4000	[60]
PRJNA604486	Treatment of PEG-6000	Paired end	Illumina NovaSeq 6000	[61]

## Data Availability

The data presented in this study are available upon request from the corresponding author and the public pomegranate transcriptomes presented in this study are available in the insert article.

## Supplementary Materials

The following supporting information can be downloaded at: https://www.mdpi.com/article/10.3390/ijms23126754/s1.
