# Supplementary material for: Systematic Identification and Expression Analysis of the Auxin Response Factor (ARF) Gene Family in Ginkgo biloba L
Source: Int J Mol Sci. 2022 Jun 17;23(12):6754. doi: 10.3390/ijms23126754 (PMC9223646; doi:10.3390/ijms23126754)
Supplement: Supplementary file 1 [file ijms-23-06754-s001.zip › ijms-1735283-supplementary/Revised supplementary materials/Table S1and S3.Detail of motifs and the secondary structure in GBARFs.pdf]

Table S1. The conserved motifs in *GbARF* family

Numbers of motifs	Numbers of amino acids	Amino acid sequences
1	50	QELVAKDLHGEEWKFRHIYRGQPRRHLLTTGWSVFVSAKRLVAGDAVJFL
2	41	PPHSFCKTLTASDTSTHGGFSVPRRAAEECFPPLDYSQQPP
3	41	LNSELWHACAGPLVSLPPVGSRVVYFPQGHMEQVAASTNQG
4	33	ERRYMGTITGIGDVDPVRWPNSKWRSLKVGWDE
5	29	PQJJCRVLNVTLHADVETDEVYAQITLVP
6	29	AAAAHAAATKSMFTVFYNPRASPSEFVIP
7	21	ALNTQLSVGMRFKMRFETEES
8	21	GWQVVYTDNEGDMMLVGDDPW
9	22	NGELRLGIRRANRQQSVMPSSV
10	22	GYEELISELARMFGJEGQLEDP
11	38	ZEFCSIVRKIFIYTPEEVZKMTPRTLSMKLSGPSZEZT
12	21	LERPERVSPWEIEPFVTPPAJ

Table S3. List of characteristic in secondary structure of GbARFs

Gene Name	Gene ID	Random coil	Alpha helix	Extended strand
GbARF3	evm.model.chr1.3000	58.38%	19.84%	21.79%
GbARF1	evm.model.chr3.2036	57.29%	24.05%	18.66%
GbARF2a	evm.model.chr4.677	53.64%	23.88%	22.47%
GbARF2b	evm.model.chr5.1456	57.30%	24.77%	17.93%
GbARF6a	evm.model.chr5.1462	**48.31%**	28.38%	23.31%
GbARF10b	evm.model.chr5.854	58.34%	18.16%	**23.50%**
GbARF19a	evm.model.chr6.93	54.48%	28.48%	17.04%
GbARF2c	evm.model.chr7.1996	59.84%	19.91%	20.25%
GbARF19b	evm.model.chr8.1790	51.07%	**35.04%**	**13.89%**
GbARF10c	evm.model.chr8.618	59.26%	18.14%	22.61%
GbARF4a	evm.model.chr10.1507	59.64%	22.45%	17.91%
GbARF10a	evm.model.chr10.178	**60.65%**	**17.06%**	22.29%
GbARF6b	evm.model.chr10.810	57.91%	25.08%	17.01%
GbARF8	evm.model.chr11.851	53.23%	29.62%	17.15%
GbARF4b	evm.model.chr11.970	49.65%	34.73%	15.62%
