# Supplementary material for: Systematic Identification and Expression Analysis of the Auxin Response Factor (ARF) Gene Family in Ginkgo biloba L
Source: Int J Mol Sci. 2022 Jun 17;23(12):6754. doi: 10.3390/ijms23126754 (PMC9223646; doi:10.3390/ijms23126754)
Supplement: Supplementary file 1 [file ijms-23-06754-s001.zip › ijms-1735283-supplementary/Revised supplementary materials/Table S7.GBARFs primer sequence.pdf]

Table S7. *GbARFs* primer sequences.

Gene name	Forward primer (5′ to 3′)	Reverse primer (5′ to 3′)
GbARF2a	TGGGTTATGAATGGTGCGGGAATG	AGCCTGTTGGTTGATGTTGTTGTTG
GbARF2b	GGTGGTCAGGTTCAAAGTGGAGATC	CTGGCTACTGGCAGAGGATTCATTG
GbARF10b	CATCGAGGAGTCAGAGCTGTCAAAC	GTTGTCACTGCCAGAGTCGGATAAG
GbARF19a	TACTATCGTTCAATGGCTGCTGCTG	GCTCTTGTTGCTGTGACTGATGTTG
GbARF2c	GTGGCTCCTGCTCCGTTGAATC	CATGGTCTTCAGTTCTTGGCATTGC
GbARF10c	ACGCAGAGTGACGCCAACAATG	CTGAACTTCCACACCTCGCCATG
GbARF4a	ACAATCAGCAGCGACCTTCTCAAG	ACTCCACCCAGTTGTCAGCAAATG
GbARF10a	CTCCCATCTTGAACGCTCTAGTGTG	ACTCCTGATTGCTGCTGCTGTTG
GbARF6b	CACTTCTCCTTGGTGATGACCCTTG	CTGCTTGTTTGCCGTTGAGTTGG
GbARF8	GCAGCAGATGACTCAGGAAGGTATG	ATCGAGCAGATGTGATGGCAGAAC
GAPDH	CTGCCAAGGCTGTAGGTAAGG	TCAGATTCCTCCTTGATGGCG
GbARF6a(ORF)	ATGCCTGTGCTGGGCCAC	TCACCTTCGTACAGTTGACTCTTCTGT
GbARF10b(ORF)	ATGGAAGAAGAATTGAATCAAAAGGGTCTGG	TTATCTTGCCATGTTGTCACTGCCA
GbARF10a(ORF)	ATGCCTGTGCCATGCAGTG	TTATCTTCCCATGTTGTCACTGCCG
