# Supplementary figures and images for: Systematic Identification and Expression Analysis of the Auxin Response Factor (ARF) Gene Family in Ginkgo biloba L
Source: Int J Mol Sci. 2022 Jun 17;23(12):6754. doi: 10.3390/ijms23126754 (PMC9223646; doi:10.3390/ijms23126754)

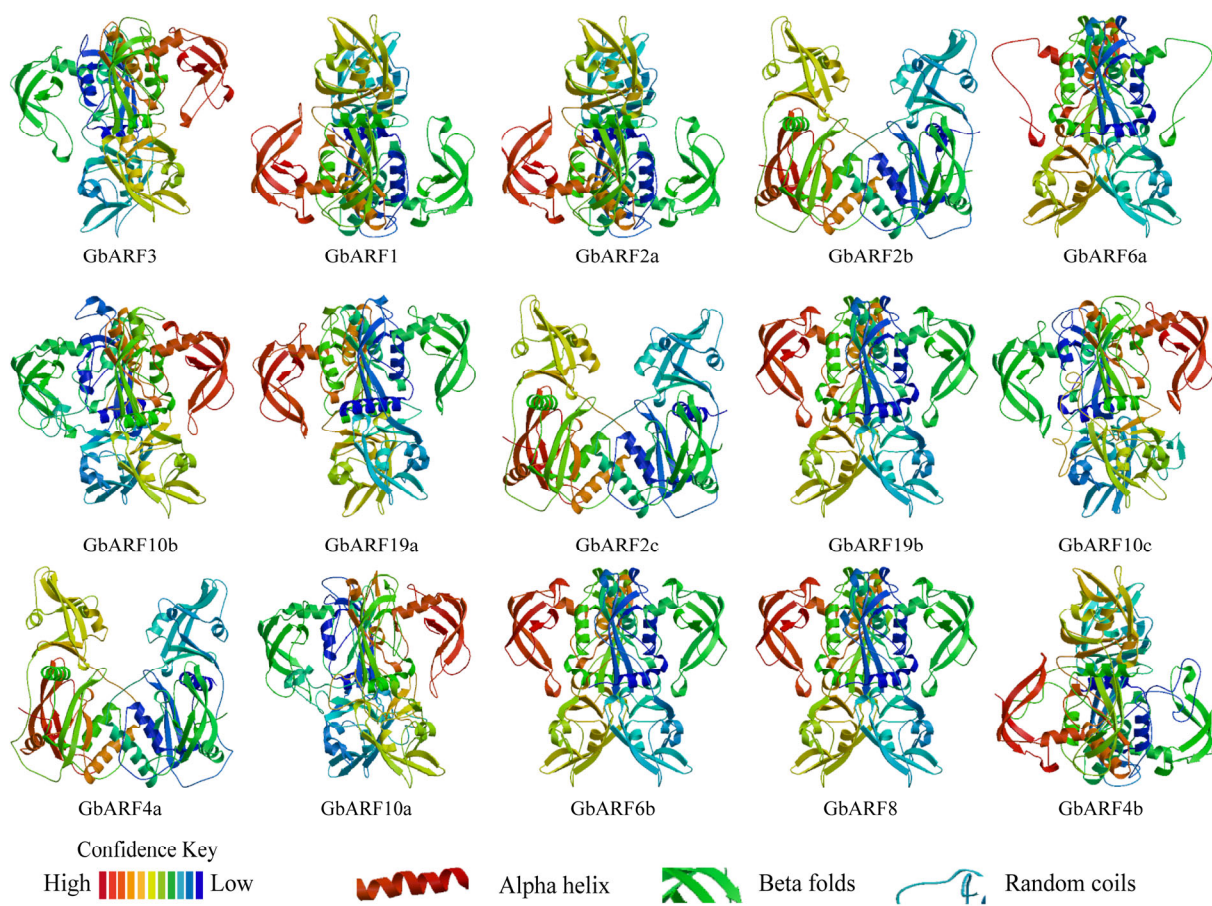

**Figure S1.** Tertiary structures of GbARF proteins

Supplement: Supplementary file 1 [file ijms-23-06754-s001.zip › ijms-1735283-supplementary/Revised supplementary materials/Figure S1.Tertiary structure of GBARF members.pdf]

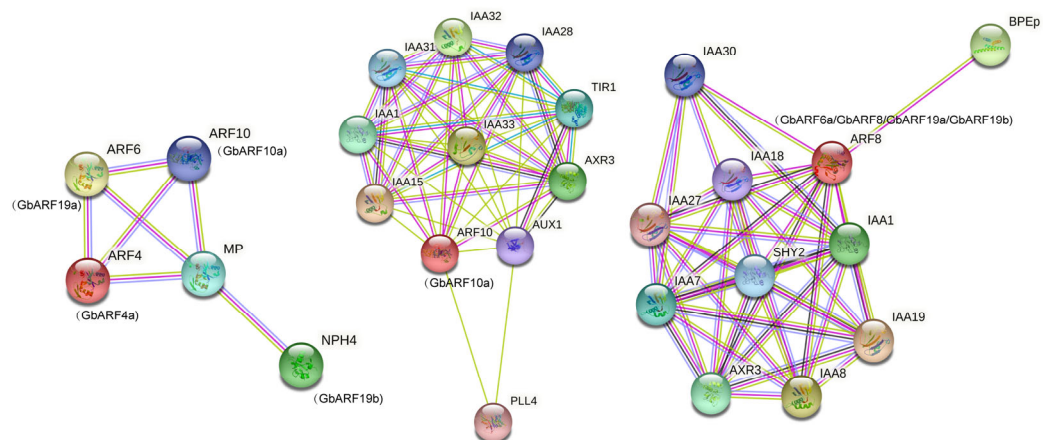

**Figure S2.** Protein interaction networks of GbARFs.

Supplement: Supplementary file 1 [file ijms-23-06754-s001.zip › ijms-1735283-supplementary/Revised supplementary materials/Figure S2.Figure 4.Protein interaction networks of GBARFs.pdf]
